# Book Reviews

**Published:** 1891-12

**Authors:** 


### Book Reviews.

---

TREATISE ON SURGERY. By C. W. Mansell Mouelin, M. D Fellow of the Royal College of Surgeons ; Surgeon to the London Hospital, etc. P. Blakiston, Son & Co., Philadelphia.

In preparing this work the author's wish has been to make it practical, and to give special attention to the question of treatment. He endeavors also to enforce the idea that the aim and object of modern surgery is to assist the tissues in their struggle against disease.

Special chapters are furnished by Mr. J. Hutchinson, Jr., on Diseases of the Eye and Skin ; by Mr. Mark Hovell, on Diseases of the Ear and Larynx ; and Mr. F. S. Eve, on Tumors.

The general pathology of surgical diseases is first discussed, constituting Part I, and this, in our opinion, is the most valuable portion of the book. It embodies the results of our latest acquired knowledge, as is shown by the statement, under *inflammation*—"mechanical irritants can only cause simple inflammation ; suppuration can not occur without pyogenic organisms; and tubercle cannot break out without its specific bacillus."

For the remainder of the book, which relates to the diseases and injuries of special organs and structures, we fail to see that it is any improvement on many other good works on surgery. It contains some new matter, of course, for instance, a very good illustrated description of Charcot's disease; but, for the most part, the subject is treated in the usual way. We doubt very much the propriety of attempting to include any longer in a work on surgery a discussion of skin diseases.

---

MINOR SURGERY AND BANDAGING. By H. R. Wharton, M. D. Surgeon to Presbyterian Hospital ; Demonstrator of Surgery in the University of Pennsylvania, etc. Lea Brothers & Co. Philadelphia.

The first ninety-eight pages of this book are devoted to a description of the various kinds of bandages, and the application of bandages to different parts of the body. In Part II. is found a full discussion of the procedures embraced by the term, *Minor Surgery*. In this part the author very properly emphasizes the importance of asepsis and antisepsis. The treatment of fractures and dislocations, the ligation of arteries and amputations are disposed of in the usual way. The book is abundantly illustrated with excellent cuts, many of which we have seen before in other places. Dr. Wharton has done his work exceedingly well, and given us a "Minor Surgery" not excelled by any other.

